# Some Difficulties with V.A.D. Workers

**Published:** 1916-04-22

**Authors:** 


*To the Editor of* The Hospital.

### Some Difficulties with V.A.D. Workers.

*To the Editor of* The Hospital.

Sir,—I sympathise greatly with "Matron, Military Hospital," in the irregularity of her V.A.D. members. I have tried several ways of organising them without success; influenza and "family affairs" make havoc of all my arrangements. I have now organised a "broom" brigade of women willing to give a few hours' service to the hospital. There are seven altogether (only four of them with Red Cross instruction), with one or two extras willing to act as understudies. They pledge themselves to send one worker morning and afternoon to cover the busiest periods, 9 to 1 and 3 to 6. They are all steady workers, so that it makes no difference to me which comes, but the leader of the brigade gives me a list every Friday of the names and hours for the ensuing week, and is pledged to fill up the time of any who may fall out unexpectedly. The workers comprise women of means, farmers' daughters, a first-rate cook, a lady's maid, and a lodging-house keeper. None of them could possibly have put in a continuous term of service, or even have come to me for alternate weeks, but they gladly give a few hours two or three times a week and are grateful to me for employing them for the wounded. I sometimes think V.A.D. work suffers because the members attempt to give more time to the hospital than they can spare from imperative home duties, with the result that constant interruptions occur.—Yours truly,

Acting Matron.

